# Decreased Brain and Placental Perfusion in Omphalopagus Conjoined Twins on Fetal MRI

**DOI:** 10.1155/2016/9458540

**Published:** 2016-03-01

**Authors:** Sureyya Burcu Gorkem, Mehmet Serdar Kutuk, Selim Doganay, Tamer Gunes, Karamehmet Yildiz, Mustafa Kucukaydin

**Affiliations:** ^1^Department of Radiology, Pediatric Radiology Division, Erciyes University School of Medicine, Kayseri, Turkey; ^2^Department of Obstetrics and Gynecology, Erciyes University School of Medicine, Kayseri, Turkey; ^3^Department of Pediatrics, Division of Neonatology, Erciyes University School of Medicine, Kayseri, Turkey; ^4^Department of Anesthesiology and Reanimation, Erciyes University School of Medicine, Kayseri, Turkey; ^5^Department of Pediatric Surgery, Erciyes University School of Medicine, Kayseri, Turkey

## Abstract

The aim of this study is to evaluate perfusional changes in brain and placenta of omphalopagus conjoined twins and to compare them with singleton fetuses by using diffusion weighted imaging and apparent diffusion coefficient. Fetal MRIs of 28-week-old omphalopagus conjoined twins with a shared liver with two separate gallbladders and portal and hepatic venous systems and three singleton fetuses with unilateral borderline ventriculomegaly at the same gestational week as control group were enrolled retrospectively. There was a significant decrease in ADC values of brain regions (*p* = 0.018) and placenta (*p* = 0.005) of conjoined twins compared to the control group. The decreased ADC values in placenta and brain regions in conjoined twins might be due to decreased placental perfusion compared to singleton pregnancy. Our results would be a keystone for future studies which will compare larger group of monochorionic multiple pregnancies with singleton pregnancies.

## 1. Introduction

Conjoined twins are a very rare congenital anomaly with the incidence of 1 per 250,000 live births [1]. The classification is based on the anatomical connection. Most common connections are thoracopagus (thorax) and omphalopagus (abdomen), where pygopagus (sacrum), ischiopagus (pelvis), craniopagus (face), or rachipagus (back) is rare. Those twins are monozygotic, monoamniotic, and monochorionic. Multiple anomalies including cardiac and circulatory anomalies, congenital diaphragm hernia, intestinal atresia, neural tube defects, or cystic hygroma can be associated [2, 3]. Omphalopagus twins account for 18–33% in which liver fuses in 80% of cases. Omphalopagus twins are joined at the front and umbilicus level; pericardium can be shared but heart is never shared [4]. Fetal MRI is superior to ultrasound for detailed imaging with complex anomalies [4, 5]. Diffusion weighted imaging (DWI) and apparent diffusion coefficient (ADC) calculations by using fetal MRI are feasible in both fetal brain and placenta in the literature [6, 7].

In this report, we aim to evaluate brain and placenta of omphalopagus conjoined twins and compare them with singleton fetuses by DWI and ADC.

## 2. Materials and Methods

**Figure 1:**
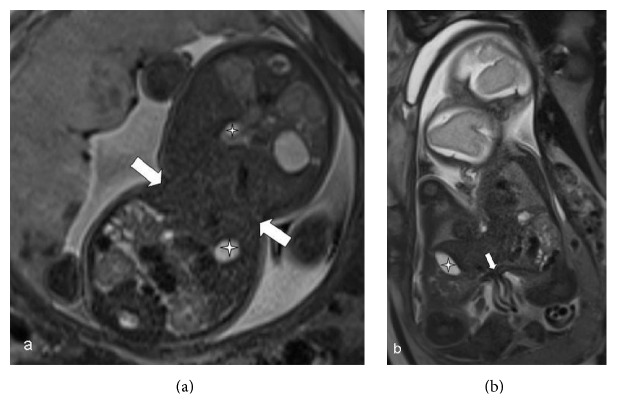
Axial (a) and coronal (b) T2-HASTE images show conjoined twins sharing liver (white arrows) at the level of umbilicus (a) with a common umbilical cord (small white arrow) (b) and two gallbladders (stars) (a, b).

**Figure 2:**
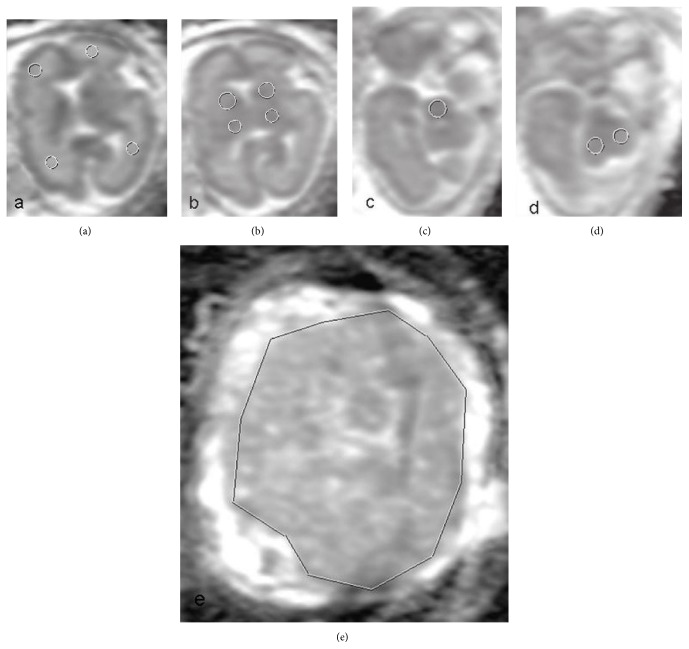
Examples of ADC calculations. ROI circles on white matter (a); thalami, lentiform nuclei (b); pons (c); cerebellum (d); and freehand ROI draw on placenta (e) are demonstrated.

Informed consent forms were taken from the patients. Fetal MRI of 28-week-old omphalopagus conjoined twins with a shared liver with two separate gallbladders and portal and hepatic venous systems and fetal MRI 28-week-old three singleton fetuses with unilateral borderline ventriculomegaly as control group were enrolled retrospectively in our study. Fetal MRI was performed following the Doppler ultrasound on the same day one or two hours later. Omphalopagus conjoined twins have single umbilical cord containing four arteries and two veins that bifurcated near the insertion site. All fetuses were appropriate for gestational age with normal Doppler findings and no growth discordance was detected [8]. Fetal MRI (1.5 T Siemens Magnetom AERA, Erlangen, Germany) procedure consisted of a three-plane scout image and three single-shot fast spin-echo sequences including T2-weighted half-fourier acquisition single-shot turbo spin-echo (HASTE) (TR/TE = 1200/94, flip angle = 150°), T1-weighted fast low angle shot magnetic resonance imaging (FLASH) (TR/TE = 169/4.76, flip angle = 70°), and T2-weighted true fast imaging with steady-state precession (TR/TE = 3.75/1.88, flip angle = 50°) in axial, sagittal, and coronal images with a slice thickness of 4 mm. Diffusion-weighted MR imaging (DWI) (*b* value = 0–1000 s/mm^2^ in three orthogonal axes (*x*, *y*, *z*)) was performed in axial for fetal brain and coronal plane for placenta as part of the routine fetal MR imaging protocol without breath holding (FOV= 320 × 320 mm, matrix = 256 × 256 mm, slice thickness = 4 mm, and time = 1 m.–24 sec.). Two pediatric radiologists with 13 (SD) and 7 (SBG) years of experience in interpreting pediatric MR images reviewed the studies together and reached a final decision in consensus. ADC-mapping was carried out on PACS (Sectra Workstation IDS7, Linköping, Sweden). ADC-map was compared with the corresponding T2-weighted images. Same circular region of interests (ROIs) was manually placed symmetrically at frontal (ROI = 0.32 cm^2^) and periatrial white matter (ROI = 0.32 cm^2^), bilateral thalami (ROI = 0.32 cm^2^), lentiform nuclei (ROI = 0.53 cm^2^), cerebellum (ROI = 0.41 cm^2^), and pons (ROI = 0.31 cm^2^) for each fetus. The DWIs of placentas were calculated by freehand ROI draws (mean = 165 cm^2^) as large as possible without risking partial volume effects which was defined along the boundary of the placental surface. Single slice calculations at the level of umbilical cord insertion on coronal image for each patient were performed and mean ADC value was taken. Regions of increased diffusion areas including vascular lakes that were hyperintense on T2-weighted images were excluded from ROI. Examples can be seen in Figures 1 and 2. Student *t*-test was used for the statistical analysis at IBM SPSS Statistics 22 (IBM SPSS Inc., Chicago, IL) and *p* < 0.05 value was considered significant.

## 3. Results

There was no diffusion restriction on both brain regions and placenta in all patients. There was no difference in ADC values of brain regions (frontal and periatrial white matter, bilateral thalami, lentiform nuclei, cerebellum, and pons) in between two conjoined fetuses (*p* = 0.453). We observed a significant decrease in ADC values of brain regions in conjoined twins (mean = 1.450 ± 0.17 × 10^−3 ^mm^2^/s) compared to control group (mean = 1.600 ± 0.19 × 10^−3 ^mm^2^/s) (*p* = 0.018). We observed a significant decrease in ADC values (*p* = 0.005) in placenta of conjoined twins (mean = 1.971 × 10^−3 ^mm^2^/s) compared to the control group (mean = 2.180 ± 0.05 × 10^−3 ^mm^2^/s) (Table 1).

Two male babies were born with a gestational weight of 1250 and 1000 grams with a caesarean section at 30 weeks of age. After two days, male babies were separated successfully. The transfontanel ultrasounds of both twins were reported to be normal. There was no evidence of hemorrhage and infarction on their ultrasound examinations. The diagnosis of common liver with two separate gallbladders and portal systems was confirmed preoperatively. They were both alive and followed up at newborn intensive care unit.

## 4. Discussion

Conjoined twin cases are rare with a female predominance and omphalopagus twins are frequently joined at the level of the umbilicus [1, 2]. Although conjoined twins are preferred to be delivered in the 36th–38th weeks by caesarean section, many are premature, as in our case. According to the fusion hypothesis of conjoined twinning, it occurs between the 13th and 15th day after fertilization, when failure to split completely leads to conjoined twins [3]. The chance of successful postnatal separation of conjoined twins depends on the complexity of fusion. Postnatal outcome depends on the degree of fusion and success of surgical approach [3]. The more the separate organs and vasculature are to be, the better the postnatal outcome and mental-motor development are [4]. For omphalopagus twins, the prenatal detection of fusion anomalies, for example, hepatobiliary tree and gastrointestinal systems, is not easy. Although prenatal ultrasound is the first line imaging modality for antenatal diagnosis, fetal MRI is more accurate to evaluate the other fetal structures and abnormalities, delivery management, and parental counseling [5].

DWI provides tissue integrity in which ADC gives the information about tissue cellularity and intact cell membrane [6]. Many factors such as cellularity, neuronal maturation, and myelination affect ADC values. Schneider et al. reported on regional differences in brain parenchyma, for example, the ADC values of supratentorial white matter higher than the ADC values of deep gray matter, cerebellum, and pons in normal fetuses [6, 7]. By gestational aging, there is a significant decline in ADC values in the cerebellum and thalamus, followed by the pons, basal ganglia, and periatrial white matter, with no significant decline in the frontal white matter due to myelination [6]. ADC calculations were done not only for detection of myelination but also for the earlier onset of perfusional changes in both brain and placenta [7]. Focal ischemic areas with low ADC values were observed in survival fetuses after death of co-twin and twin to twin transfusion in the literature [9, 10]. Recent studies showed decreased placental perfusion in IUGR by using DWI and perfusion mapping in human and animal models [7, 11–14]. There is a developmental delay in twins comparing normal singleton fetuses [15]. As a result of sharing the same placenta, monochorionic twins can be more complicated to IUGR due to inadequate circulation than singleton pregnancy. Anatomically shared organs and circulations make conjoined twins more vulnerable to perfusional problems than monochorionic twins [15]. The results of our study show that decreased ADC values in placenta and brain regions in conjoined twins might be due to decreased placental perfusion compared to singleton pregnancy. There are no reported nomogram of ADC values for twins in the literature. Therefore, our study is the first in the literature which calculated ADC values of brain and placenta in conjoined twins with normal Doppler findings and showed perfusion differences in between conjoined twins and normal singleton fetuses although they do not show any diffusion restriction.

Our study provides information about omphalopagus conjoined twins by showing decreased brain and placental perfusion which may result in developmental delay compared to singleton fetuses at the same gestational week. We believe that our results would be a keystone for future studies which will compare larger group of monochorionic multiple pregnancies with singleton pregnancies.

## Figures and Tables

**Table 1:** Fetal brain and placenta ADC values (mm^2^/s).

Fetal regions	(I) fetus^*∗*^	(II) fetus^*∗*^	Control group^*∗*^	*p*
Frontal WM	1.688	1.687	1.850 ± 0.03	0.003
Periatrial WM	1.659	1.647	1.850 ± 0.04	0.004
Thalamus	1.315	1.318	1.450 ± 0.04	0.014
Lentiform nuclei	1.337	1.385	1.500 ± 0.01	0.003
Cerebellum	1.420	1.452	1.580 ± 0.01	0.005
Pons	1.221	1.229	1.370 ± 0.03	0.004
Placenta	1.971		2.180 ± 0.05	0.005

^*∗*^Mean ADC = ×10^−3^ mm^2^/s.

WM: white matter.
